# HILPDA is a lipotoxic marker in adipocytes that mediates the autocrine negative feedback regulation of triglyceride hydrolysis by fatty acids and alleviates cellular lipotoxic stress

**DOI:** 10.1016/j.molmet.2023.101773

**Published:** 2023-07-06

**Authors:** Lei Deng, Shuangcheng Alivia Wu, Ling Qi, Sander Kersten

**Affiliations:** 1 Nutrition, Metabolism and Genomics Group, Division of Human Nutrition and Health, Wageningen University, the Netherlands; 2 Department of Molecular & Integrative Physiology, University of Michigan Medical School, Ann Arbor, MI, 48105, USA; 3 Division of Metabolism, Endocrinology and Diabetes, Department of Internal Medicine, University of Michigan Medical School, Ann Arbor, MI, 48105, USA; 4 Division of Nutritional Sciences, Cornell University, Ithaca, NY, 14853, USA

**Keywords:** HILPDA, Fatty acids, ATGL, ER stress, Autocrine negative feedback, Adipocytes

## Abstract

**Background:**

Lipolysis is a key metabolic pathway in adipocytes that renders stored triglycerides available for use by other cells and tissues. Non-esterified fatty acids (NEFAs) are known to exert feedback inhibition on adipocyte lipolysis, but the underlying mechanisms have only partly been elucidated. An essential enzyme in adipocyte lipolysis is ATGL. Here, we examined the role of the ATGL inhibitor HILPDA in the negative feedback regulation of adipocyte lipolysis by fatty acids.

**Methods:**

We exposed wild-type, HILPDA-deficient and HILPDA-overexpressing adipocytes and mice to various treatments. HILPDA and ATGL protein levels were determined by Western blot. ER stress was assessed by measuring the expression of marker genes and proteins. Lipolysis was studied in vitro and in vivo by measuring NEFA and glycerol levels.

**Results:**

We show that HILPDA mediates a fatty acid-induced autocrine feedback loop in which elevated intra- or extracellular fatty acids levels upregulate HILPDA by activation of the ER stress response and the fatty acid receptor 4 (FFAR4). The increased HILPDA levels in turn downregulate ATGL protein levels to suppress intracellular lipolysis, thereby maintaining lipid homeostasis. The deficiency of HILPDA under conditions of excessive fatty acid load disrupts this chain of events, leading to elevated lipotoxic stress in adipocytes.

**Conclusion:**

Our data indicate that HILPDA is a lipotoxic marker in adipocytes that mediates a negative feedback regulation of lipolysis by fatty acids via ATGL and alleviates cellular lipotoxic stress.

## Highlights

• HILPDA is a central node in a fatty acid-induced autocrine feedback loop in adipocytes.
• HILPDA is activated by the unfolded protein response and the fatty acid receptor 4 (FFAR4).
• The deficiency of HILPDA leads to more ER stress under conditions of increased fatty acid load.
• HILPDA suppresses ATGL protein levels and intracellular lipolysis in response to fatty acid overload.

## 1 Introduction

The main function of adipose tissue is to store excess energy as triglycerides. An average human adult carries sufficient amounts of triglycerides to survive at least 4 weeks of complete food deprivation. The amount of triglycerides stored in the adipose tissue is determined by the balance between triglyceride synthesis and triglyceride hydrolysis (lipolysis), e.g. triglyceride turnover. Estimates of the daily turnover rate of triglycerides for an average-sized adult vary from 50 to 100 g/d [[1], [2], [3]].

The fatty acids used to synthesize triglycerides in adipose tissue are mainly derived from the circulating triglyceride-rich lipoproteins VLDL and chylomicrons. The triglycerides in these lipoproteins are hydrolyzed by the enzyme lipoprotein lipase (LPL), which is secreted by adipocytes and subsequently delivered to the endothelial surface [4]. After uptake by adipocytes, the fatty acids are transported to the endoplasmic reticulum where they are esterified to triglycerides through the sequential addition of fatty acyl moieties to a glycerol-3-phosphate backbone. The last, and reportedly rate-limiting, step in triglyceride synthesis involves the addition of acyl-CoA to diacylglycerol and is catalyzed by diacylglycerol acyltransferase (DGAT). Two evolutionarily distinct DGAT isoenzymes exist, DGAT1 and DGAT2 [5]. It was shown that DGAT1 and DGAT2 can largely compensate for each other to support triglyceride storage in adipocytes [6]. However, DGAT1 is unique in being able to protect the ER from the lipotoxic effects of high-fat diets [7]. Following DGAT-catalyzed triglyceride synthesis, the lipids are stored in a single large lipid droplet surrounded by a phospholipid monolayer and decorated with numerous lipid-droplet-associated proteins.

As part of regular triglyceride turnover, a portion of the triglycerides stored in lipid droplets is hydrolyzed to fatty acids. The sequential removal of fatty acids from the triglyceride molecule is catalyzed by the enzymes adipose triglyceride lipase (ATGL), hormone-sensitive lipase, and monoglyceride lipase [8]. A portion of the liberated fatty acids is secreted and ends up in the bloodstream, accounting for most of the non-esterified fatty acid (NEFA) pool in the plasma. The other part is re-esterified to triglycerides in the adipocyte [9]. Since glycerol released by lipolysis cannot be re-used by the adipocyte, the molar ratio of fatty acids to glycerol released provides an estimate of the relative rate of fatty acid re-esterification. In weight-stable, never-obese control subjects, this ratio of NEFA to glycerol leaving the adipocytes was reported to be 1.4, suggesting that under baseline conditions most of the fatty acids are re-esterified [10].

Adipose tissue lipolysis is under tight hormonal control [11,12]. Cortisol, (nor)epinephrine, and growth hormone stimulate the activity of lipolytic enzymes, whereas insulin has the opposite effect. The effects of metabolic hormones on lipolytic enzymes are mainly mediated by post-translational mechanisms and concentrate on ATGL, which is considered rate-limiting for lipolysis [[13], [14], [15]]. In addition to activation via PKA and AMPK-mediated phosphorylation, ATGL is regulated through the physical interaction with (in)activating proteins such as CGI-58 and G0S2. CGI-58, also known as ABHD5, is a catalytically inactive member of the family of α/β hydrolase domain-containing proteins that binds and activates ATGL [16], whereas G0S2 is an inhibitor of ATGL (Yang et al., 2010). A more recently identified co-regulatory protein of ATGL is HILPDA [17]. HILPDA (Hypoxia-Induced Lipid Droplet Associated) is a small lipid droplet-associated protein that is expressed in macrophages, hepatocytes, cancer cells, and adipocytes [18]. The levels of HILPDA are increased by various stimuli including hypoxia, β-adrenergic activation, and fatty acids. Consistent with the ability of ATGL to bind to and inhibit ATGL [19,20], gain and loss of function studies have shown that HILPDA promotes triglyceride accumulation in hepatocytes [[21], [22], [23]], macrophages [[24], [25], [26]], and cancer cells [20,27]. Currently, the physiological role of HILPDA in adipose tissue is not fully clear [28,29]. Previous studies did not reveal a clear effect of adipocyte-specific HILPDA deficiency on in vivo lipolysis under conditions of fasting, cold exposure, or β3-adrenergic activation [28].

Besides hormonal cues, lipolysis is regulated by fatty acids, which are the products of the lipolytic reaction [[30], [31], [32]]. Recently, it was reported that the fatty acid receptor 4 (FFAR4) plays a role in mediating the feedback inhibition by fatty acids on lipolysis [33,34]. Here we explored the role of HILPDA in the feedback inhibition of adipocyte lipolysis by fatty acids by studying 1) the regulation of HILPDA by fatty acids, 2) the regulation of ATGL by HILPDA, and 3) the functional impact of HILPDA deficiency in vitro and in vivo. We show that HILPDA mediates a fatty acid-induced autocrine feedback loop in adipocytes in which elevated intra- or extracellular fatty acid levels upregulate HILPDA levels by activation of the unfolded protein response and FFAR4, which in turn suppresses ATGL-catalyzed intracellular triglyceride hydrolysis to maintain lipid homeostasis and prevent lipotoxicity.

## 2 Experimental procedures

### 2.1 Animal study

#### 2.1.1 Mice and diets

*Hilpda*^flox/flox^ mice (Jackson Laboratories, Bar Harbor, ME; Hilpda^tm1.1Nat^/J, RRID: IMSR_JAX:017,360) were crossed with Adiponectin-Cre transgenic mice (Jackson Laboratories, Bar Harbor, ME; B6·FVB-Tg (Adipoq-cre)1Evdr/J, RRID: IMSR_JAX:028,020) and backcrossed onto a C57BL/6J background in our facility for at least 5 generations. *Hilpda*^flox/flox^ mice are characterized by LoxP sites flanking the second exon of *Hilpda*, followed by the open reading frame for membrane-tethered human placental alkaline phosphatase (ALPP) after the second loxP site. Following Cre recombination, ALPP is expressed under the control of the *Hilpda* promoter. *Hilpda*^flox/flox^ mice were crossed with *Hilpda*^flox/flox^ mice heterozygous for Adiponectin-Cre, yielding 50% *Hilpda*^flox/flox^ and 50% adipocyte-specific HILPDA-deficient (*Hilpda*^ΔADIPO^) mice, equally distributed among males and females. The *Hilpda*^flox/flox^ and *Hilpda*^ΔADIPO^ mice used in the studies were littermates.

Mice were group housed at 21-22 °C under specific pathogen-free conditions and a 6:00–18:00 day–night cycle. Mice had ad libitum access to regular chow and water unless otherwise indicated.

For fasting/refeeding, male *Hilpda*^ΔADIPO^ and *Hilpda*^flox/flox^ mice aged 4–5 months were subjected to 24 h of fasting or 20 h of fasting followed by 4 h of refeeding with chow. Water was available ad libitum during the entire period of fasting/refeeding. The number of mice per group was 9–13.

For low-fat/high-fat feeding, male *Hilpda*^ΔADIPO^ and *Hilpda*^flox/flox^ mice aged 10–13 weeks were randomly allocated using an online randomization tool to either a standardized high-fat diet or low-fat diet (formula D12451 and formula D12450H respectively, Research Diets Inc., New Brunswick, USA; γ-irradiated with 10–20 kGy) for 20 weeks. During this period, the mice were housed individually in type 2 cages. Body weight and food intake were assessed weekly. The number of mice per group was 9–12. After 16 weeks of high-fat feeding, an intraperitoneal glucose tolerance test was carried out.

At the end of both studies, the mice were anesthetized with isoflurane. Blood was collected via orbital puncture in tubes containing EDTA (Sarstedt, Nümbrecht, Germany). Immediately thereafter, mice were euthanized by cervical dislocation, after which tissues were excised, weighed, and frozen in liquid nitrogen or prepared for histology. Frozen samples were stored at −80 °C.

All animal experiments were approved by the Institutional Animal Care and Use Committee of Wageningen University (AVD104002015236; 2016. W-0093.002, 2016. W-0093.007).

#### 2.1.2 Intraperitoneal glucose tolerance test

Mice were moved to fresh cages without food 5 h before the glucose tolerance test. Blood was collected via tail bleeding for baseline blood glucose measurement. Immediately thereafter, the mice received an intraperitoneal injection of glucose at 1 g/kg body weight, followed by blood collection via tail bleeding at 15, 30, 45, 60, 90, and 120 min. Blood glucose was measured with a GLUCOFIX Tech glucometer and glucose sensor test strips (GLUCOFIX Tech, Menarini Diagnostics, Valkenswaard, the Netherlands).

#### 2.1.3 Plasma measurements

Blood collected in EDTA tubes (Sarstedt, Numbrecht, Germany) was centrifuged for 10 min at 2,000 g at 4 °C. Plasma was collected, aliquoted, and stored at −80 °C. After thawing, plasma was analyzed for cholesterol (Liquicolor, Human GmbH, Wiesbaden, Germany), triglycerides (Liquicolor), glucose (Liquicolor), glycerol (Liquicolor), NEFAs (NEFA-HR set R1, R2 and standard, WAKO Diagnostics, Instruchemie, Delfzijl, The Netherlands), adiponectin (ELISA duoset kit, R&D Systems, Bio-techne, MN, USA), leptin (ELISA duoset kit, R&D Systems) and insulin (ultra-sensitive mouse insulin ELISA kit, Crystal Chem Inc., IL, USA) following the manufacturer's instructions.

#### 2.1.4 Liver triglyceride measurement

Two-percent liver homogenates were prepared in buffer (10 mM Tris, 2 mM EDTA and 0.25 M sucrose, pH 7.5) using a Tissue Lyser II (Qiagen, Hilden, Germany). Liver triglyceride content was quantified using a Triglyceride LiquiColor mono kit from HUMAN Diagnostics (Wiesbaden, Germany) according to the manufacturer's instructions.

### 2.2 Cell culture

#### 2.2.1 3T3-L1 adipocytes

3T3-L1 fibroblasts were amplified in DMEM supplemented with 10% FCS and 1% penicillin/streptomycin (culture medium) and subsequently seeded into the desired plates (15,000 cells/cm^2^). Two days after the cells reached confluence, the medium was changed to DMEM supplemented with 10% FCS containing 0.5 mM 3-isobutyl-1-methylxanthine (Sigma-Aldrich; I5879), 2 μg/ml insulin (Sigma-Aldrich; I9278), 0.5 μM dexamethasone (Sigma-Aldrich; D4902), and 1 μM rosiglitazone (Sigma-Aldrich; R2408). After 3 days, the medium was changed to culture medium supplemented with 2 μg/ml insulin and 1 μM rosiglitazone until the cells were fully differentiated.

#### 2.2.2 Mouse SVF-derived adipocytes

Adipocytes were differentiated from the stromal vascular fraction (SVF), which was obtained from inguinal white adipose tissue of *Hilpda*^ΔADIPO^ and *Hilpda*^flox/flox^ mice. Briefly, dissected adipose tissue depots were kept and cleaned in an ice-cooled transport medium (DMEM plus 1% fatty acid-free BSA (Sigma-Aldrich)). Cleaned adipose tissue samples were minced into small pieces and incubated with collagenase solution (DMEM, 3.2 mM CaCl2, 15 mM HEPES, 0.5% BSA, 10% FCS, and 1.5 mg/ml collagenase type II (Sigma-Aldrich; C6885)) at 37 °C for 30 min. The digested tissue suspensions were then filtered using a 100-μm cell strainer and centrifuged at 300 g for 10 min at room temperature. The pellet stromal vascular fractions were resuspended and grown in cell culture flasks until around 90% confluency. Cells were seeded in the culture plate with a density of 15,000 cells/cm^2^ in DMEM supplemented with 10% FCS and 1% penicillin/streptomycin. Two to 3 days post-seeding (at full confluency), differentiation was started by supplementing with 0.5 mM of 3-isobutyl-1-methylxanthine (Sigma-Aldrich; I5879), 1 mM of dexamethasone (Sigma-Aldrich; D4902), 7 mg/ml of human insulin (Sigma-Aldrich; I2643), and 1 μM of rosiglitazone (Sigma-Aldrich; R2408). After 3 days of stimulation, cells were further cultured in insulin medium (DMEM containing 7 mg/ml human insulin) for another 3 days followed by a normal growth medium (DMEM, 10% FCS, and 1% penicillin/streptomycin).

For the comparative mRNA analysis between SVF and adipocytes, floating adipocytes were collected and snap-frozen for RNA isolation. The pelleted SVF was resuspended in TRIzol (Thermo Fisher Scientific, Landsmeer, The Netherlands) and snap-frozen for RNA isolation.

#### 2.2.3 Fatty acid treatment (OA:PA)

A 2:1 mixture of oleate (Sigma-Aldrich; P0500) and palmitate (Sigma-Aldrich; O1008) was added to mature mouse SVF-derived adipocytes or 3T3-L1 adipocytes. Fatty acids were dissolved in ethanol and diluted with 70 mM KOH to a 25 mM stock solution for cell culture application.

#### 2.2.4 Agonist, antagonist, and inhibitor assays

Mature mouse SVF-derived adipocytes or 3T3-L1 adipocytes were treated with 10 μM isoproterenol (Sigma-Aldrich; I6504) for 3 h or 10 μM forskolin (Sigma-Aldrich; F6886) for 2 h to stimulate lipolysis. Cells were treated with 20 μM DGAT1 inhibitor T863 (Sigma-Aldrich; AML0539) and/or 10 μM DGAT2 inhibitor PF-06424439 (Merck; PZ0233) for the indicated time durations to inhibit cellular DGAT1 and DGAT2, respectively. Atglistatin (50 μM, Sigma-Aldrich; 5.30151) was used to inhibit ATGL, and GW9662 (5 nM, Tocris; 1508) was used as a PPARγ antagonist in combination with the treatment of the cells with free fatty acids. AH6714 (10 μM, Tocris; 5256) was used as an antagonist of GPR120/FFAR4. 4μ8c (10 μM, Tocris; 4479), ceapin A7 (10 μM, Tocris; 6955), and AMG PERK 44 (10 μM, Tocris; 5517) were used to inhibit the IRE1α, ATF6, and PERK branches of the UPR pathway, respectively. MG132 (10 μM, Abcam; ab141003) was used to inhibit proteasomal degradation, and leupeptin (10 μM, Sigma-Aldrich; L2884) to inhibit lysosomal degradation. Etomoxir (20 μM, Sigma-Aldrich; E1905) was used to inhibit mitochondrial fatty acid oxidation, and 10,12-Tricosadiynoic acid (1 μM, Sigma-Aldrich; 91445) to inhibit peroxisomal fatty acid oxidation. The treatments were performed during days 7–10 of the differentiation. The antagonists and inhibitors were applied to cells 30 min before the treatments with OA:PA, isoproterenol, or forskolin. After pre-incubation, cells were washed with culture medium twice. After the treatment, the medium was collected for glycerol and NEFA analysis using relevant kits (Instruchemie, The Netherlands), following the manufacturer's protocols. The cells were washed with PBS twice before further processing to isolate protein or mRNA.

For the lipolysis assay, as described previously [28], mature mouse SVF-derived adipocytes (*Hilpda*^ΔADIPO^ and *Hilpda*^flox/flox^) were serum starved for 2 h in DMEM with 1% fatty acid–free BSA and subsequently treated with 10 μM isoproterenol for 3 h in 1% fatty acid–free BSA supplemented DMEM medium. After this, the medium was collected for NEFA measurement.

#### 2.2.5 Adenovirus transduction

3T3-L1 cells were transduced with an MOI of 500 PFU 3 days after inducing differentiation. Specifically, after dilution with 0.5 μg/ml poly-l-lysine (prepared in DMEM only) and incubation at 37 °C for 60 min, adenoviruses were added to serum-starved cells at an MOI of 500 PFU. Each well in the 24-well plate was filled with 100 μL virus-supplemented medium. After 1.5 h of incubation, 200 μL medium supplemented with 10% FCS was added and the transduction continued for a total of 24 h. Starting from this time point and till the end of the transduction, 7 μg/ml insulin was supplemented to the medium for cell differentiation. Thereafter, the medium was changed to the normal culture medium (DMEM containing 10% FCS and 1% penicillin/streptomycin) and cells were cultured for another 48 h. Ad-m-Hig2 (ADV-250639) was purchased from Vector Biolabs.

### 2.3 Quantitative RT-PCR

Total RNA was isolated using TRizol Reagent (Thermo Fisher Scientific, 15596018). cDNA was synthesized using the iScript cDNA Synthesis Kit (Bio-Rad Laboratories, Inc., 1708890) following the manufacturer's protocol. Real-Time polymerase chain reaction (RT-PCR) was performed on the CFX 384 Touch Real-Time detection system (Bio-Rad Laboratories, Inc., California, United States), using the SensiMix (BioLine, BIO-83005) protocol for SYBR green reactions. Mouse *36B4* expression was used for normalization.

### 2.4 RT-PCR for Xbp1 splicing

RT-PCR was performed on cDNA as previously described [35]. Briefly, the PCR products were amplified at an annealing temperature of 58 °C for 33 cycles, and then were separated by electrophoresis on a 2.5% agarose gel. Images were acquired using the ChemiDoc MP system (Bio-Rad Laboratories, Inc., United States).

### 2.5 Immunoblotting

The cell lysates were prepared using RIPA Lysis and Extraction Buffer (Thermo Fisher Scientific, 89901) supplemented with protease inhibitor (Thermo Fisher Scientific; A32965) and phosphatase inhibitor (Roche; 4906845001) and quantified with Pierce BCA Protein Assay Kit (Thermo Fisher Scientific, Massachusetts, United States). The gonadal white adipose tissue homogenates were prepared in the same buffer by Tissue Lyser II. The fat was removed by centrifuging 3 times at 11,000 rpm for 10 min at 4 °C. The protein lysates were separated by electrophoresis on pre-cast 4–15% polyacrylamide gels and transferred onto nitrocellulose membranes using a Trans-Blot Semi-Dry transfer cell (Bio-Rad Laboratories, Inc., California, United States). The membranes were blocked in 5% skim milk in TBS-T (TBS buffer supplied with 1‰ TWEEN 20) and incubated with HILPDA antibody (Rabbit antisera against amino acid resides 37–64 of murine HILPDA generated by Pineda, Berlin, Germany), ATGL antibody (Cell Signaling Technology; 2138), and HSP90 antibody (Cell Signaling Technology; 4874) overnight at 4 °C. Secondary antibody incubations were performed at room temperature for 1 h. Images were acquired using the ChemiDoc MP system (Bio-Rad Laboratories, Inc., United States). Protein analysis of ER stress markers on fasting and refed mouse adipose tissue was performed at the University of Michigan according to the methods previously described [35]. Specifically, for Phos-tag gel running, 5% SDS-PAGE containing 50 μM Phos-tag (NARD Institute) and 50 μM MnCl2 (Sigma) was applied. Afterward, gels were kept in 1 mM EDTA for 10 min before transferring. The intensity of each band was quantitated using densitometry analysis, normalized against HSP90, and indicated relative to the control condition.

### 2.6 Statistical analysis

Data were analyzed using unpaired Student's t-test or two-way ANOVA followed by Tukey's multiple comparisons test. A value of p < 0.05 was considered statistically significant. Details are presented in the figure legends.

## 3 Results

### 3.1 Regulation of HILPDA in adipocytes by extracellular fatty acids

**Figure 1:**
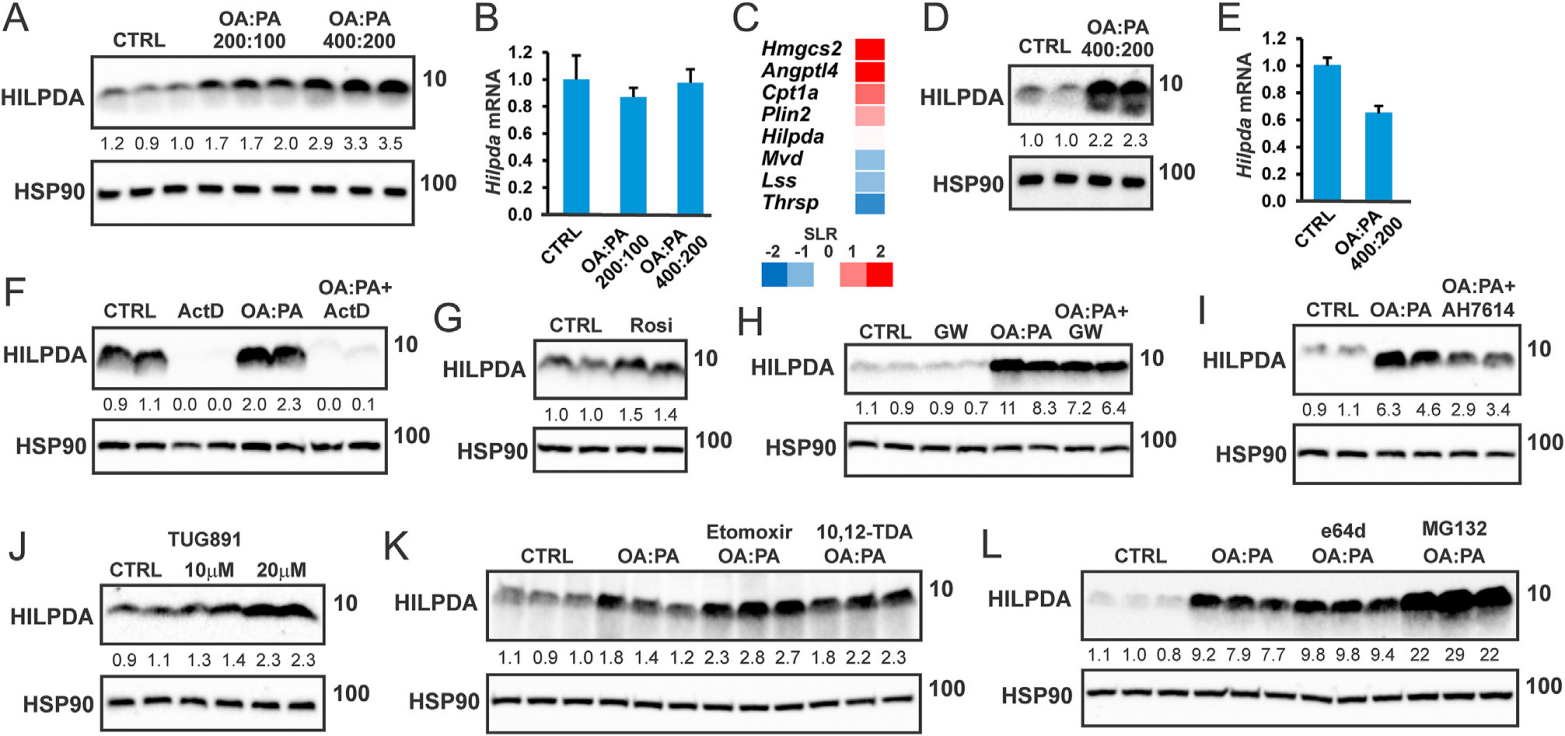
**External fatty acids induce HILPDA expression in adipocytes partly via FFAR4**. A). HILPDA protein level and B) mRNA expression of *Hilpda* in 3T3-L1 adipocytes treated with a 2:1 mixture of oleate and palmitate (OA:PA, concentrations are indicated in μM) for 12 h. N = 3. C) Lipid-sensitive gene expression in 3T3-L1 adipocytes treated with 1 mM oleate for 24 h. SLR, signal log ratio. D) HILPDA protein level and E) mRNA expression of *Hilpda* in SVF-derived adipocytes treated with OA:PA (600 μM) for 12 h. N = 3. F) HILPDA protein level in SVF-derived adipocytes treated with OA:PA (600 μM) in the presence or absence of 10 μM actinomycin D for 6 h. HILPDA protein level in SVF-derived adipocytes treated with G) 10 μM rosiglitazone, H) 600 μM OA:PA in the presence or absence of 5 nM PPARγ antagonist GW9662, or I) 10 μM FFAR4 antagonist AH7614 for 12 h. J) HILPDA protein level in SVF-derived adipocytes treated with TUG-891 for 24 h. HILPDA protein level in 3T3-L1 adipocytes treated with 600 μM OA:PA in the presence or absence of K) 20 μM CPT1 inhibitor etomoxir or 1 μM ACOX1 inhibitor 10,12-Tricosadiynoic acid, or L) 10 μM lysosomal protease inhibitor e64d or proteasomal protease inhibitor MG132 for 12 h. The cells were preincubated with inhibitors for 30 min before treatment with OA:PA. Western blots were probed with antibodies against HILPDA and HSP90.

To examine the regulation of HILPDA by fatty acids in adipocytes, we treated mouse 3T3-L1 adipocytes with a 2:1 mixture of oleic acid and palmitic acid (OA:PA). As observed in hepatocytes, macrophages, and mouse embryonic stem cells [21,24,26,27], OA:PA treatment dose-dependently increased HILPDA protein levels (Figure 1A). The increase in HILPDA protein by OA:PA was not accompanied by any change in *Hilpda* mRNA (Figure 1B), indicating that fatty acids induce HILPDA at the post-transcriptional level. Unlike *Hilpda*, the mRNA expression of *Angptl4*, *Cpt1a*, and *Hmgcs2* were increased by oleic acid in 3T3-L1 adipocytes (Figure 1C). Similar to the observation in 3T3-L1 adipocytes, OA:PA upregulated HILPDA protein but not mRNA levels in SVF-derived adipocytes (Figure 1D,E). The induction of HILPDA by OA:PA was maintained when DNA transcription was blocked by Actinomycin D, despite a decrease in baseline HILPDA protein levels (Figure 1F). These data underscore the rapid turnover of HILPDA and suggest that the induction of HILPDA protein by fatty acids in adipocytes is not mediated by increased HILPDA transcription.

Fatty acids are known to activate PPARγ [36,37]. Since the expression of *Hilpda* is controlled by PPARγ [28], fatty acids might increase HILPDA protein via PPARγ activation. In line with HILDPA being a PPARγ target, HILPDA protein levels in adipocytes were induced by the PPARγ agonist Rosiglitazone (Figure 1G). However, the induction of HILPDA protein by OA:PA was only modestly affected by the PPARγ antagonist GW9662 (Figure 1H), suggesting that fatty acids upregulate HILPDA in adipocytes mostly independently of PPARγ.

Fatty acids also activate the cell surface receptor FFAR4 (GPR120) [38]. Accordingly, we hypothesized that fatty acids may upregulate HILPDA via the activation of FFAR4. In line with this notion, the increase in HILPDA protein by OA:PA was attenuated by the FFAR4 antagonist AH7614 (Figure 1I). In addition, the FFAR4 agonist TUG-891 increased HILPDA protein in SVF-derived adipocytes (Figure 1J). Together, these data suggest that the stimulatory effect of fatty acids on HILPDA levels in adipocytes is partly dependent on FFAR4.

The induction of HILPDA by fatty acids was enhanced by the mitochondrial fatty acid oxidation inhibitor etomoxir, and to a lesser extent by the peroxisomal fatty acid oxidation inhibitor 10,12-Tricosadiynoic acid (Figure 1K). Finally, the induction of HILPDA by OA:PA could be further enhanced by the co-treatment with the proteasomal inhibitor MG132 but not by co-treatment with e64d (Figure 1L), which inhibits lysosomal proteases and interferes with autolysosomal digestion, suggesting that HILPDA is broken down via proteasomal degradation but not lysosomal degradation.

### 3.2 Regulation of HILPDA in adipocytes by intracellular fatty acids

**Figure 2:**
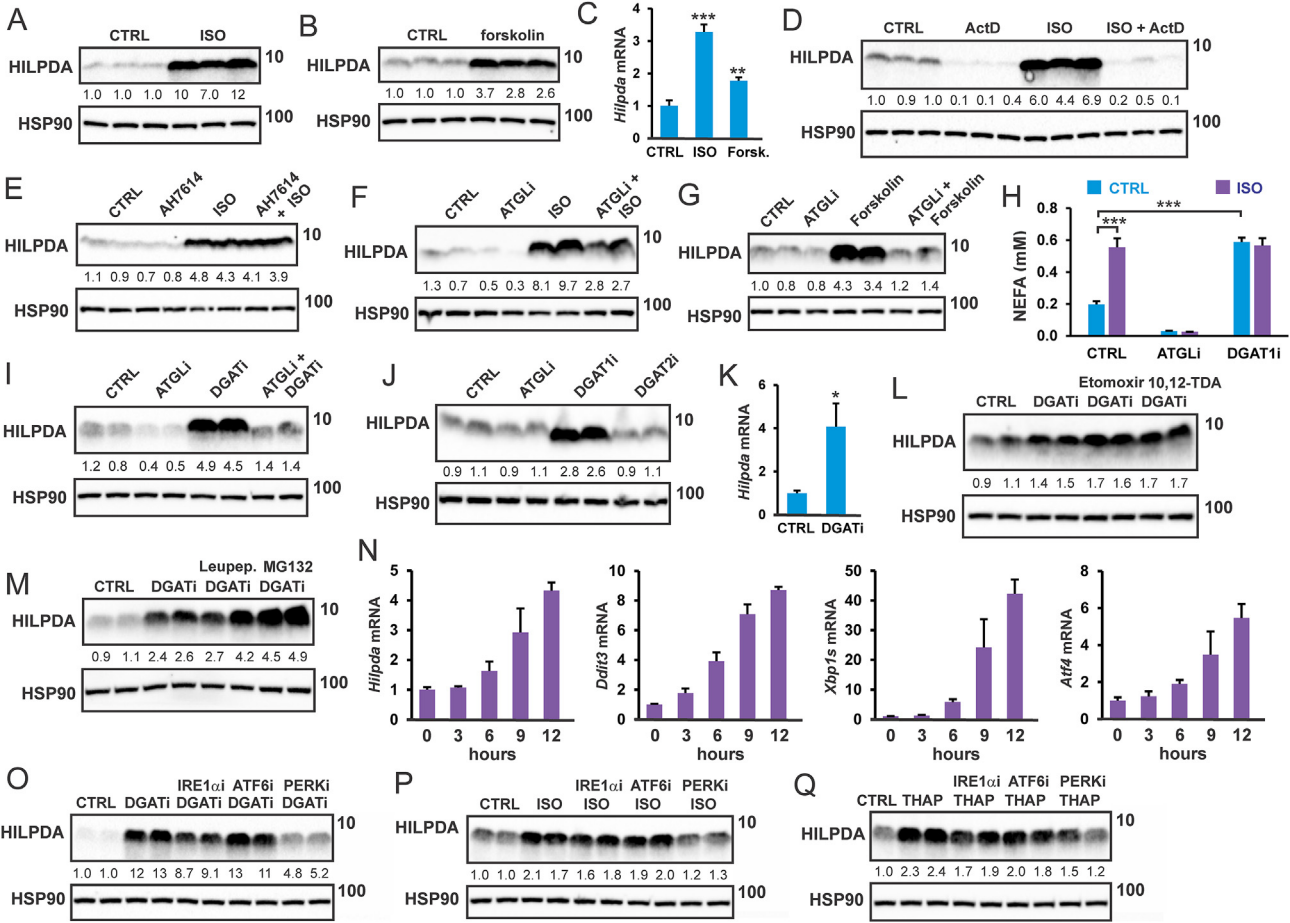
**Intracellular fatty acids induce HILPDA in adipocytes via ER stress**. HILPDA protein level in SVF-derived adipocytes treated with A) 10 μM isoproterenol and B) forskolin for 3 h. C) *Hilpda* mRNA level after treatments. HILPDA protein level in SVF-derived adipocytes treated for 3 h with 10 μM isoproterenol in the presence and absence of D) 10 μM actinomycin D or E) 10 μM AH7614. HILPDA protein level in SVF-derived adipocytes treated with F) 10 μM isoproterenol for 3 h or G) 10 μM forskolin for 2 h, in the presence or absence of 50 μM Atglistatin (ATGLi). H) NEFA levels in medium of SVF-derived adipocytes after treatment with 10 μM isoproterenol, in the presence or absence of 50 μM Atglistatin (ATGLi) or 20 μM T863 (DGAT1i) for 3 h. N = 3. I) HILPDA protein level in SVF-derived adipocytes treated with 20 μM T863 (DGAT1i) and 10 μM PF-06424439 (DGAT2i) for 10 h in the presence or absence of 50 μM Atglistatin. J) HILPDA protein level in SVF-derived adipocytes treated with 50 μM Atglistatin, 20 μM T863 (DGAT1i), or 10 μM PF-06424439 (DGAT2i) for 10 h. K) *Hilpda* mRNA expression in SVF-derived adipocytes treated with DGAT1/DGAT2 inhibitors for 10 h. N = 3. HILPDA protein level in SVF-derived adipocytes treated with DGAT1/DGAT2 inhibitors for 10 h in the presence or absence of L) 10 μM etomoxir or 1 μM 10,12-tricosadiynoic acid, or M) 10 μM leupeptin or MG132. N) mRNA expression of *Hilpda*, *Ddit3*, *Xbp1s* and *Atf4* in 3T3-L1 adipocytes treated with DGAT1/DGAT2 inhibitors for different durations. N = 3. HILPDA protein level in 3T3-L1 adipocytes treated with O) DGAT1/DGAT2 inhibitors for 10 h, P) 10 μM isoproterenol for 3 h, and Q) 5 μM thapsigargin for 12 h, in the presence or absence of specific ER stress pathway inhibitors. Western blots were probed with antibodies against HILPDA and HSP90. Asterisk indicates significantly different from control treatment according to Student's t-test. ∗P < 0.05, ∗∗P < 0.01, ∗∗∗P < 0.001.

Previously, we showed that isoproterenol and forskolin increase HILPDA levels in 3T3-L1 adipocytes [28]. However, we were unable to identify the mechanism. Similar to 3T3-L1 adipocytes, isoproterenol and forskolin increased HILPDA protein levels in SVF-derived mouse adipocytes (Figure 2A,B). The induction of HILPDA protein by forskolin and isoproterenol was accompanied by increased *Hilpda* mRNA (Figure 2C). HILPDA induction by isoproterenol was blocked by actinomycin D (Figure 2D), but was not affected by the FFAR4 antagonist AH7614 (Figure 2E). Interestingly, the induction of HILPDA by isoproterenol was partially abolished by ATGL inhibition (Figure 2F), while the induction of HILPDA by forskolin was completely abolished by ATGL inhibition (Figure 2G). These data point to a crucial role of lipolysis and intracellular fatty acids in the induction of HILPDA by forskolin and isoproterenol.

Another way to increase the intracellular levels of fatty acids is via inhibition of fatty acid esterification by chemical inhibition of DGAT (DGATi), which subsequently leads to enhanced release of fatty acids by adipocytes (Figure 2H). Consistent with the stimulation of HILPDA by intracellular fatty acids, DGATi causes a pronounced increase in HILPDA protein levels (Figure 2I), which was abolished by ATGL inhibition. The stimulatory effect of DGATi on HILPDA levels could be attributed to the inhibition of DGAT1 (Figure 2J). The induction of HILPDA protein by DGATi was accompanied by increased *Hilpda* mRNA (Figure 2K) and was further enhanced by the chemical inhibition of fatty acid oxidation (Figure 2L) and inhibition of proteasomal degradation (Figure 2M). Interestingly, when DGAT was chemically inhibited, isoproterenol failed to increase NEFA release by adipocytes, suggesting that isoproterenol mainly inhibits DGAT-mediated fatty acid re-esterification (Figure 2H).

Previously, DGAT inactivation was shown to lead to ER stress in adipocytes [7]. Supporting this finding, DGAT inhibition increased the expression of ER stress marker genes (Figure 2N). Accordingly, we hypothesized that raising intracellular fatty acids by DGAT inhibition and isoproterenol may induce HILPDA by triggering ER stress and subsequent activation of the unfolded protein response (UPR). To verify this notion, we treated adipocytes with DGATi or isoproterenol in conjunction with inhibitors of different UPR branches. Notably, induction of HILPDA by DGATi was attenuated by inhibition of PERK, while inhibition of IRE1α signaling modestly suppressed the induction of HILPDA (Figure 2O). Similarly, induction of HILPDA by isoproterenol was attenuated by PERK inhibition (Figure 2P). Supporting a stimulatory effect of ER stress on HILPDA, treatment of adipocytes with the ER stressor Thapsigargin increased HILPDA protein levels, which again was attenuated by PERK inhibition (Figure 2Q). These data suggest that the elevation of intracellular fatty acid levels raises HILPDA expression at least partly by triggering ER stress, which increases HILPDA levels mainly via activation of the PERK/eIF2α signaling branch of UPR.

### 3.3 Regulation of ATGL in adipocytes by HILPDA

**Figure 3:**
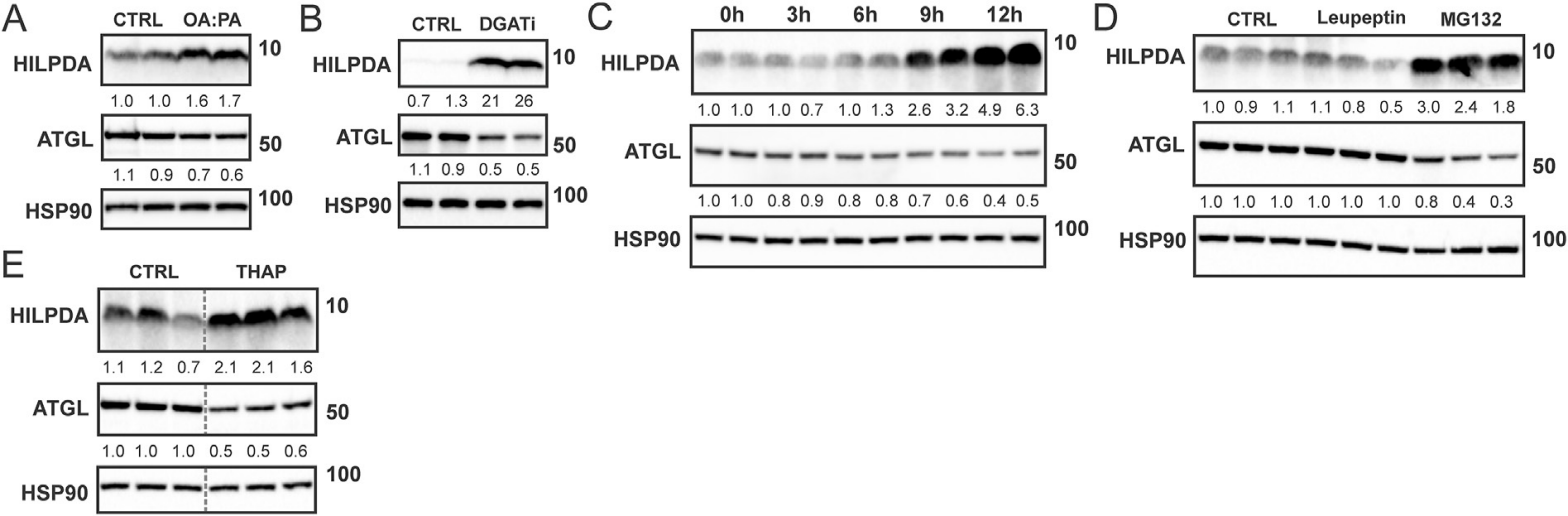
**ATGL protein levels in adipocytes are inversely associated with HILPDA protein levels**. Protein levels of HILPDA and ATGL in SVF-derived adipocytes treated with A) 600 μM OA:PA for 12 h, or B) DGAT1/DGAT2 inhibitors for 10 h. 3T3-L1 adipocytes were treated with C) DGAT1/DGAT2 inhibitors for different durations, or D) 10 μM leupeptin or MG132 for 14 h, or E) 5 μM thapsigargin for 12 h. The grey dotted line indicates that lanes were not adjacent but pasted from different sections of the gel. Western blots were probed with antibodies against HILPDA, ATGL, and HSP90.

HILPDA is an inhibitor of ATGL [19,20]. Interestingly, data from macrophages suggest that under certain conditions, the interaction between HILPDA and ATGL leads to a reduction in ATGL protein levels [25,26]. Accordingly, we hypothesized that the induction of HILPDA by fatty acids may be associated with a decrease in ATGL protein. Consistent with this notion, treatment of SVF-derived adipocytes with OA:PA (Figure 3A) or DGATi (Figure 3B) increased HILPDA levels in parallel with a decrease in ATGL protein levels. A time-course experiment of DGAT inhibition showed that ATGL levels start to drop when HILPDA levels increase (Figure 3C). The concurrent induction of HILPDA and reduction in ATGL were also observed upon treatment of adipocytes with the proteasomal inhibitor MG132 (Figure 3D) or ER stressor Thapsigargin (Figure 3E). These data show that induction of HILPDA by fatty acids and ER stress is paralleled by a reduction in ATGL protein levels.

**Figure 4:**
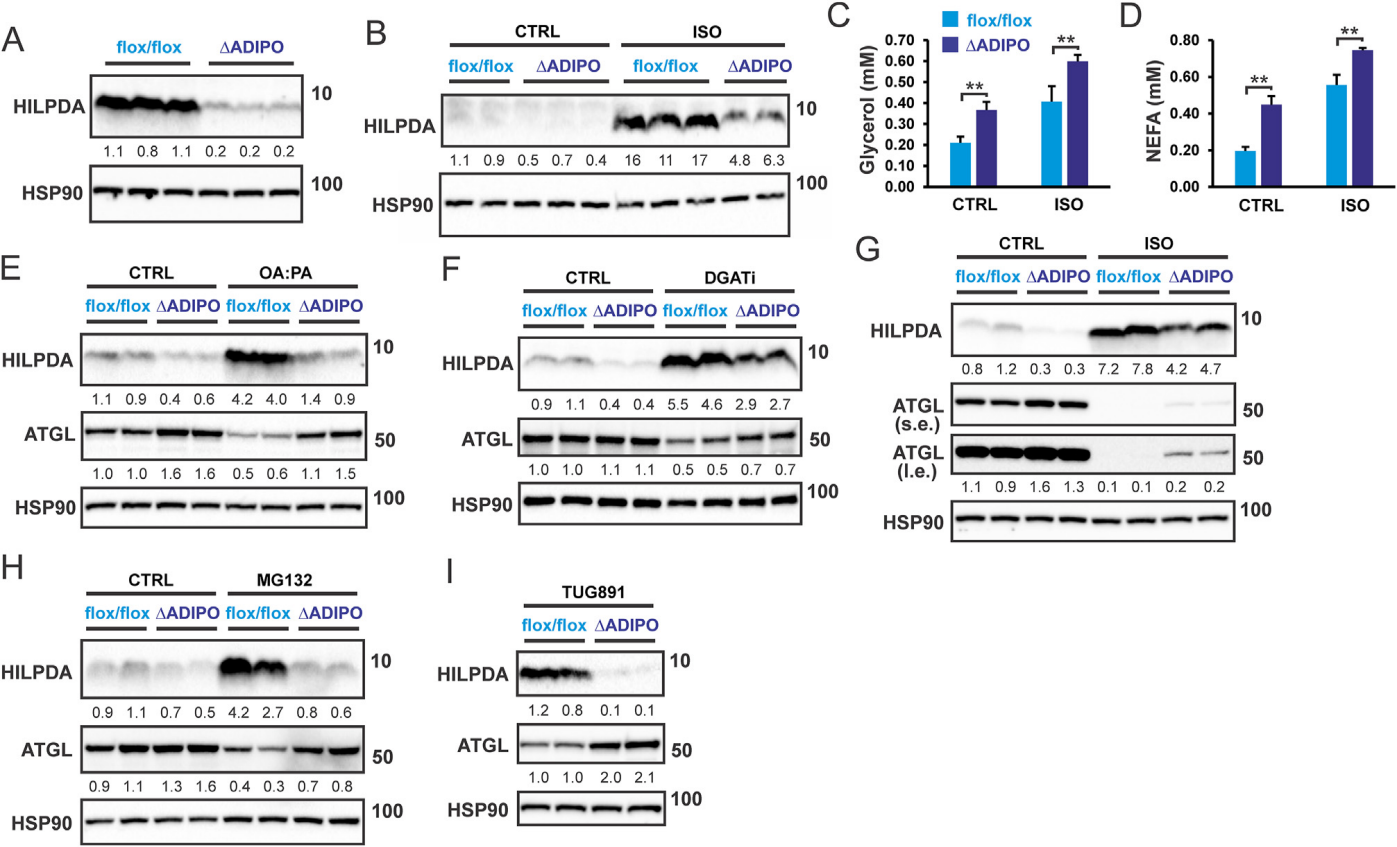
**HILPDA reduces ATGL protein levels in adipocytes**. A) HILPDA protein level in *Hilpda*^flox/flox^*and Hilpda*^ΔADIPO^ SVF-derived adipocytes, B) HILPDA protein level in *Hilpda*^flox/flox^ and *Hilpda*^ΔADIPO^ SVF-derived adipocytes treated with 10 μM isoproterenol for 3 h. C) Free glycerol and D) NEFA concentration in the culture medium of *Hilpda*^flox/flox^ and *Hilpda*^ΔADIPO^ SVF-derived adipocytes treated with 10 μM isoproterenol for 3 h. N = 3. E) HILPDA and ATGL protein levels in *Hilpda*^flox/flox^ and *Hilpda*^ΔADIPO^ SVF-derived adipocytes treated with E) 600 μM OA: PA for 12 h, F) DGAT1/DGAT2 inhibitors for 2 h, G) 10 μM isoproterenol for 3 h, H) 10 μM MG132 for 14 h, or I) 20 μM TUG-891 for 24 h. L. e., long exposure; s. e., short exposure. Western blots were probed with antibodies against HILPDA, ATGL, and HSP90. Asterisk indicates significantly different according to Student's t-test. ∗∗P < 0.01.

To investigate if the increase in HILPDA is responsible for the decrease in ATGL protein levels upon elevation of extra- and intracellular fatty acids, we used SVF-derived adipocytes obtained from adipocyte-specific HILPDA-deficient mice (*Hilpda*^ΔADIPO^) and control mice (*Hilpda*^flox/flox^). HILPDA protein levels, either in the basal state (Figure 4A) or after treatment with isoproterenol (Figure 4B), were strongly reduced in *Hilpda*^ΔADIPO^ adipocytes compared to *Hilpda*^flox/flox^ adipocytes. Supporting the inhibitory effect of HILPDA on ATGL-mediated intracellular lipolysis, the release of glycerol (Figure 4C) and NEFA (Figure 4D) was significantly higher in adipocytes obtained from *Hilpda*^ΔADIPO^ mice compared to *Hilpda*^flox/flox^ mice. In agreement with the suppression of ATGL protein levels by HILPDA, ATGL protein levels were higher in *Hilpda*^ΔADIPO^ adipocytes compared to *Hilpda*^flox/flox^ adipocytes treated with OA:PA (Figure 4E), DGATi (Figure 4F), or isoproterenol (Figure 4G). Similarly, ATGL protein levels were higher in *Hilpda*^ΔADIPO^ adipocytes compared to *Hilpda*^flox/flox^ adipocytes treated with MG132 or TUG-891 (Figure 4H,I), concurrent with higher levels of HILPDA. The results of these experiments indicate that the induction of HILPDA at least partially mediates the decrease in adipocyte ATGL levels upon elevation of extra- and intracellular fatty acid levels.

### 3.4 HILPDA deficiency leads to enhanced ER stress under conditions of fatty acid overload

**Figure 5:**
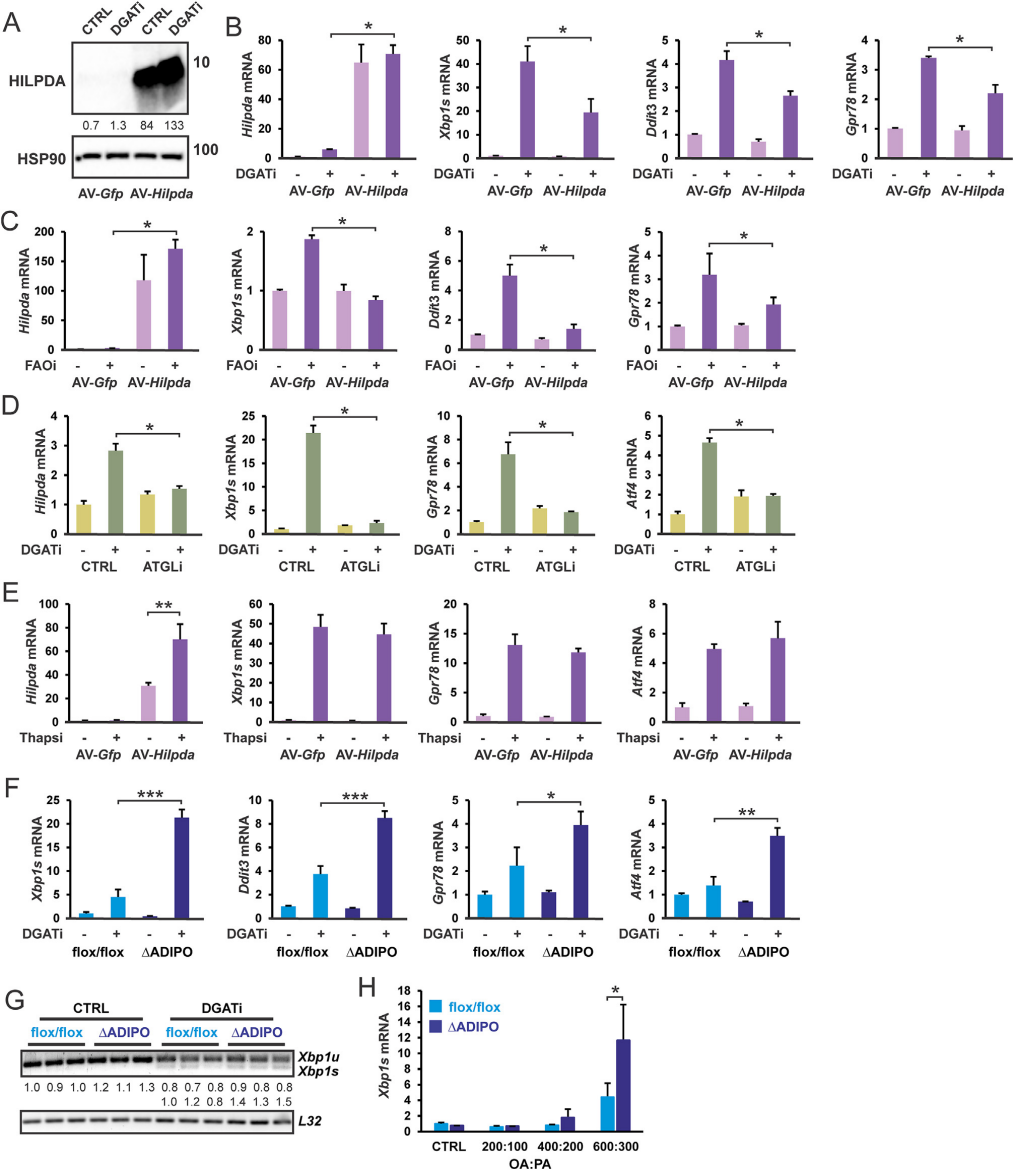
**HILPDA protects adipocytes from fatty acid-induced ER stress**. A) HILPDA protein level in AV-*Hilpda* transduced 3T3-L1 cells. Western blot was probed with antibodies against HILPDA and HSP90. mRNA expression of *Hilpda* and ER stress markers in AV-*Hilpda* transduced 3T3-L1 cells treated with B) DGAT1/DGAT2 inhibitors for 10 h, and C) 600 μM OA:PA combined with 20 μM etomoxir and 1 μM 10,12- tricosadiynoic acid for 12 h. D) mRNA expression of *Hilpda* and ER stress markers in SVF-derived adipocytes treated with DGAT1/DGAT2 inhibitors for 10 h in the presence and absence of 50 μM Atglistatin. E) mRNA expression of *Hilpda* and ER stress markers in AV-*Hilpda* transduced 3T3-L1 adipocytes treated with 5 μM thapsigargin for 14 h. F) mRNA expression in *Hilpda*^flox/flox^ and *Hilpda*^ΔADIPO^ SVF-derived adipocytes treated with DGAT1/DGAT2 inhibitors for 10 h. G) mRNA expression of spliced and unspliced *Xbp1* in *Hilpda*^flox/flox^ and *Hilpda*^ΔADIPO^ SVF-derived adipocytes treated with DGAT1/DGAT2 inhibitors for 10 h. H) mRNA expression of spliced *Xbp1s* in *Hilpda*^flox/flox^ and *Hilpda*^ΔADIPO^ SVF-derived adipocytes treated with 300 μM, 600 μM, or 900 μM OA:PA for 24 h. Asterisk indicates significantly different according to Student's t-test. ∗P < 0.05, ∗∗P < 0.01, ∗∗∗P < 0.001.

The collective data presented so far are suggestive of a feedback mechanism in which fatty acid overload may inhibit the generation of additional fatty acids by suppressing intracellular triglyceride lipolysis by downregulating ATGL protein levels via induction of HILPDA. As indicated above, the elevation of intracellular fatty acid levels can induce ER stress and the UPR pathway. To investigate if HILPDA may protect against fatty acid-induced ER stress, we overexpressed HILPDA in 3T3-L1 adipocytes using an adenovirus (Figure 5A), which was previously shown to reduce ATGL protein levels [28]. As expected, raising intracellular fatty acid levels, either by inhibiting DGAT (Figure 5B) or fatty acid oxidation (Figure 5C), increased the expression of ER stress marker genes. Interestingly, this increase was attenuated by HILPDA overexpression. The effect of HILPDA overexpression on ER stress marker genes was less pronounced compared to the chemical inhibition of ATGL (Figure 5D), suggesting that HILPDA overexpression does not fully inactivate ATGL. As a negative control, the induction of ER stress marker genes by Thapsigargin was unaffected by HILPDA overexpression (Figure 5E). These data suggest that HILPDA overexpression protects against lipotoxicity. Conversely, to examine if deficiency of HILPDA may exacerbate fatty acid-induced ER stress, we measured the expression of ER stress marker genes in *Hilpda*^ΔADIPO^ and *Hilpda*^flox/flox^ adipocytes treated with DGAT inhibitors. ER stress markers were higher in *Hilpda*^ΔADIPO^ adipocytes treated with DGAT inhibitors compared to *Hilpda*^flox/flox^ adipocytes (Figure 5F). DGAT inhibition also increased the levels of spliced *Xbp1* mRNA relative to unspliced *Xbp1*, which was minimally enhanced in *Hilpda*^ΔADIPO^ adipocytes (Figure 5G). These data suggest that under conditions of elevated intracellular fatty acids, HILPDA deficiency removes the restriction on ATGL-mediated lipolysis, enhancing intracellular fatty acid overload and ER stress. Similarly, HILPDA deficiency was associated with elevated spliced *Xbp1* mRNA in adipocytes treated with OA:PA but only at very high concentrations of fatty acids (Figure 5H). Collectively, the data support the notion that HILPDA attenuates lipotoxicity under conditions of fatty acid overload by suppressing ATGL-mediated lipolysis.

### 3.5 HILPDA deficiency increases markers of ER stress in adipose tissue in vivo

**Figure 6:**
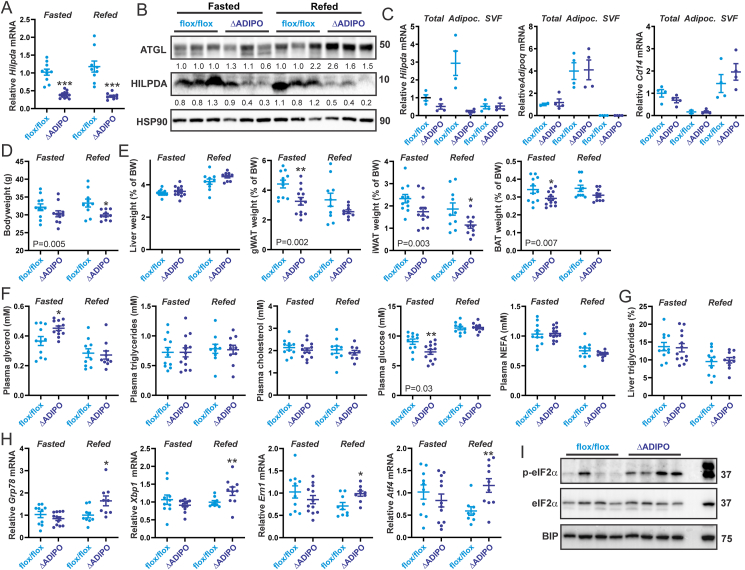
**HILPDA alleviates ER stress in adipose tissue during refeeding**. A) Relative *Hilpda* mRNA expression in epididymal white adipose tissue of *Hilpda*^flox/flox^ and *Hilpda*^ΔADIPO^ mice after 24 h of fasting or 20 h of fasting followed by 4 h of refeeding with chow (n = 9–13 per group). B) HILPDA, ATGL, and HSP90 protein levels in epididymal white adipose tissue. C) mRNA levels of *Hilpda*, *Adipoq* (adipocyte marker), and *Cd14* (endothelial marker) in epididymal white adipose tissue, freshly separated adipocytes, and the stromal vascular fraction of *Hilpda*^flox/flox^ (n = 4) and *Hilpda*^ΔADIPO^ mice (n = 4). Gene expression levels of *Hilpda* in adipose tissue from *Hilpda*^flox/flox^ mice were set at one. D) Bodyweight of *Hilpda*^flox/flox^ and *Hilpda*^ΔADIPO^ mice after 24 h of fasting or 20 h of fasting followed by 4 h of refeeding with chow (n = 9–13 per group). gWAT, gonadal (epididymal) adipose tissue; iWAT, inguinal adipose tissue; BAT, brown adipose tissue. E) Weight of the liver and various adipose tissue depots. F) Plasma metabolites. G) Liver triglyceride content (%, wt/wt). H) Relative expression of ER stress genes in epididymal white adipose tissue. I) Protein levels of eIF2α, phosphorylated eIF2α, and BIP in epididymal white adipose tissue from fasted/refed *Hilpda*^flox/flox^ and *Hilpda*^ΔADIPO^ mice. Right lane is positive control of mouse adipocytes treated with thapsigargin. Western blots were probed with antibodies against eIF2α, p-eIF2α, BIP, HILPDA and HSP90. In the graphs, the horizontal bar represents the mean and the error bars represent SEM. P values in the figures reflect the statistical significance of the comparison *Hilpda*^flox/flox^ versus *Hilpda*^ΔADIPO^ by two-way ANOVA. Asterisk indicates significantly different from *Hilpda*^flox/flox^ mice according to Tukey's posthoc test. ∗P < 0.05, ∗∗P < 0.01, ∗∗∗P < 0.001.

A physiological condition associated with increased lipolysis and increased fatty acid flux in adipocytes is fasting. Accordingly, to investigate whether HILPDA maintains intracellular fatty acid homeostasis in vivo, *Hilpda*^ΔADIPO^ and *Hilpda*^flox/flox^ mice were subjected to a 24 h fast or to a 20 h fast followed by a 4 h refeed. In both the fasted and refed groups, *Hilpda* mRNA (Figure 6A) and protein levels (Figure 6B) in adipose tissue were lower in the *Hilpda*^ΔADIPO^ mice compared to *Hilpda*^flox/flox^ mice. The lower *Hilpda* expression in whole adipose tissue of *Hilpda*^ΔADIPO^ mice could be attributed to a reduction in *Hilpda* mRNA in the adipocyte as opposed to the stromal vascular fraction (Figure 6C). As observed in adipocytes treated with DGATi, HILPDA deficiency in adipocytes was accompanied by increased ATGL protein content (Figure 6B), which was specifically observed after fasting/refeeding. These data suggest that HILPDA influences ATGL protein levels in adipose tissue but only under specific metabolic conditions.

Further analysis of the phenotype showed that body weight was significantly lower in the *Hilpda*^ΔADIPO^ mice than in the *Hilpda*^flox/flox^ mice (Figure 6D), as was the relative weight of the gonadal, inguinal, and subscapular brown adipose tissue depot (Figure 6E). By contrast, relative liver weight was unaffected by HILPDA deficiency (Figure 6E). To examine the metabolic effects of HILPDA deficiency in fasted and refed mice, several plasma metabolites were measured. Interestingly, plasma glycerol levels were modestly but significantly elevated in fasted *Hilpda*^ΔADIPO^ compared to *Hilpda*^flox/flox^ mice (Figure 6F). By contrast, plasma cholesterol, triglycerides, and NEFA levels were similar in *Hilpda*^ΔADIPO^ and *Hilpda*^flox/flox^ mice after either fasting or fasting/refeeding (Figure 6F). Intriguingly, plasma glucose levels were significantly lower in the *Hilpda*^ΔADIPO^ compared to *Hilpda*^flox/flox^ mice in the fasted state (Figure 6F). The elevation in plasma glycerol levels in fasted *Hilpda*^ΔADIPO^ mice suggest an increase in adipose tissue lipolysis, which in turn might account for the lower weight of various adipose depots. Liver triglyceride content was not significantly different between the *Hilpda*^ΔADIPO^ and *Hilpda*^flox/flox^ mice (Figure 6G).

To examine if HILPDA deficiency might influence the sensitivity of adipose tissue to fatty acid-induced stress, we measured the expression of ER stress marker genes in gonadal adipose tissue of fasted and fasted/refed *Hilpda*^ΔADIPO^ and *Hilpda*^flox/flox^ mice. Adipose tissue mRNA levels of *Grp78*, *Xbp1*, *Atf4*, and *Ern1* were modestly but significantly higher in *Hilpda*^ΔADIPO^ mice than in *Hilpda*^flox/flox^ mice, which was specifically observed after fasting/refeeding (Figure 6H). Further analysis of the activation of the different branches of the UPR pathway by Western blot showed increased eIF2α phosphorylation in the adipose tissue of *Hilpda*^ΔADIPO^ mice after fasting/refeeding (Figure 6I). By contrast, other UPR markers were either not different between *Hilpda*^ΔADIPO^ and *Hilpda*^flox/flox^ mice (BIP, Figure 6I) or were not well detectable in murine adipose tissue (CHOP, (phosphorylated) IRE1α, not shown). These data are indicative of increased ER stress in the adipose tissue of *Hilpda*^ΔADIPO^ mice after fasting/refeeding and support the notion that the UPR pathway mainly targeted by elevated fatty acids is eIF2α.

**Figure 7:**
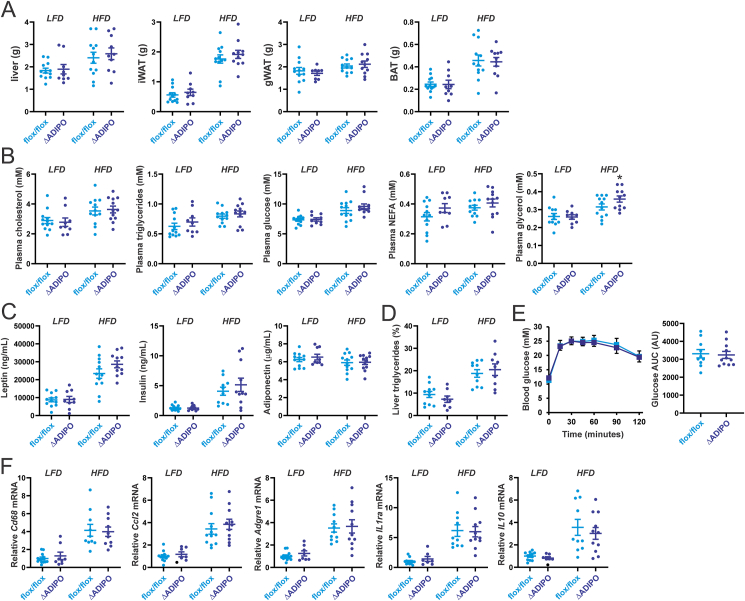
**HILPDA does not influence HFD-induced metabolic and inflammatory complications.** A) Weight of the liver and various adipose tissue depots in *Hilpda*^flox/flox^ and *Hilpda*^ΔADIPO^ mice fed a low-fat diet (LFD) or high-fat diet (HFD) for 20 weeks (n = 9–12 per group). gWAT, gonadal (epididymal) adipose tissue; iWAT, inguinal adipose tissue; BAT, brown adipose tissue. B) Plasma concentration of glucose, glycerol, triglycerides, cholesterol, and NEFA. C) Plasma levels of metabolism-related hormones. D) Liver triglyceride content (%, wt/wt). E) Intraperitoneal glucose tolerance test and Area Under the Curve. F) Relative mRNA expression of inflammation-related genes in epididymal white adipose tissue. In the graphs, the horizontal bar represents the mean and the error bars represent SEM. Asterisk indicates significantly different from *Hilpda*^flox/flox^ mice according to Tukey's posthoc test. ∗P < 0.05.

Apart from fasting/refeeding, another physiological condition that is associated with altered fatty acid flux in adipose tissue is high-fat feeding. Accordingly, we fed *Hilpda*^ΔADIPO^ mice and *Hilpda*^flox/flox^ mice a high-fat diet for 20 weeks, using a low-fat diet as control. The weights of the liver, gonadal adipose tissue, inguinal adipose tissue, and brown adipose tissue were not significantly different between the two genotypes on either the low-fat diet or high-fat diet (Figure 7A). Furthermore, plasma cholesterol, triglycerides, glucose, and NEFA levels were similar in *Hilpda*^ΔADIPO^ and *Hilpda*^flox/flox^ mice on the low-fat or high-fat diet (Figure 7B). By contrast, after high-fat feeding, plasma glycerol levels were modestly but significantly elevated in *Hilpda*^ΔADIPO^ compared to *Hilpda*^flox/flox^ mice (Figure 7B), suggesting that adipocyte HILPDA deficiency leads to increased adipose tissue lipolysis during high-fat feeding.

Further analysis of the metabolic phenotype did not reveal any differences between *Hilpda*^ΔADIPO^ and *Hilpda*^flox/flox^ mice. Specifically, plasma leptin, adiponectin, and insulin (Figure 7C), as well as hepatic triglyceride levels (Figure 7D) were not significantly different between *Hilpda*^ΔADIPO^ and *Hilpda*^flox/flox^ mice on either diet. Furthermore, glucose tolerance was not significantly affected by HILPDA deficiency in the high-fat diet group (Figure 7E). Gene expression analysis showed increased expression of inflammation-related genes in the adipose tissue of mice fed the high-fat diet compared to mice fed the low-fat diet but no differences were observed between *Hilpda*^ΔADIPO^ and *Hilpda*^flox/flox^ mice (Figure 7F). Collectively, these data show that except for a modest increase in plasma glycerol, adipocyte HILPDA deficiency did not significantly influence metabolic parameters in mice fed a high-fat diet.

## 4 Discussion

**Figure 8:**
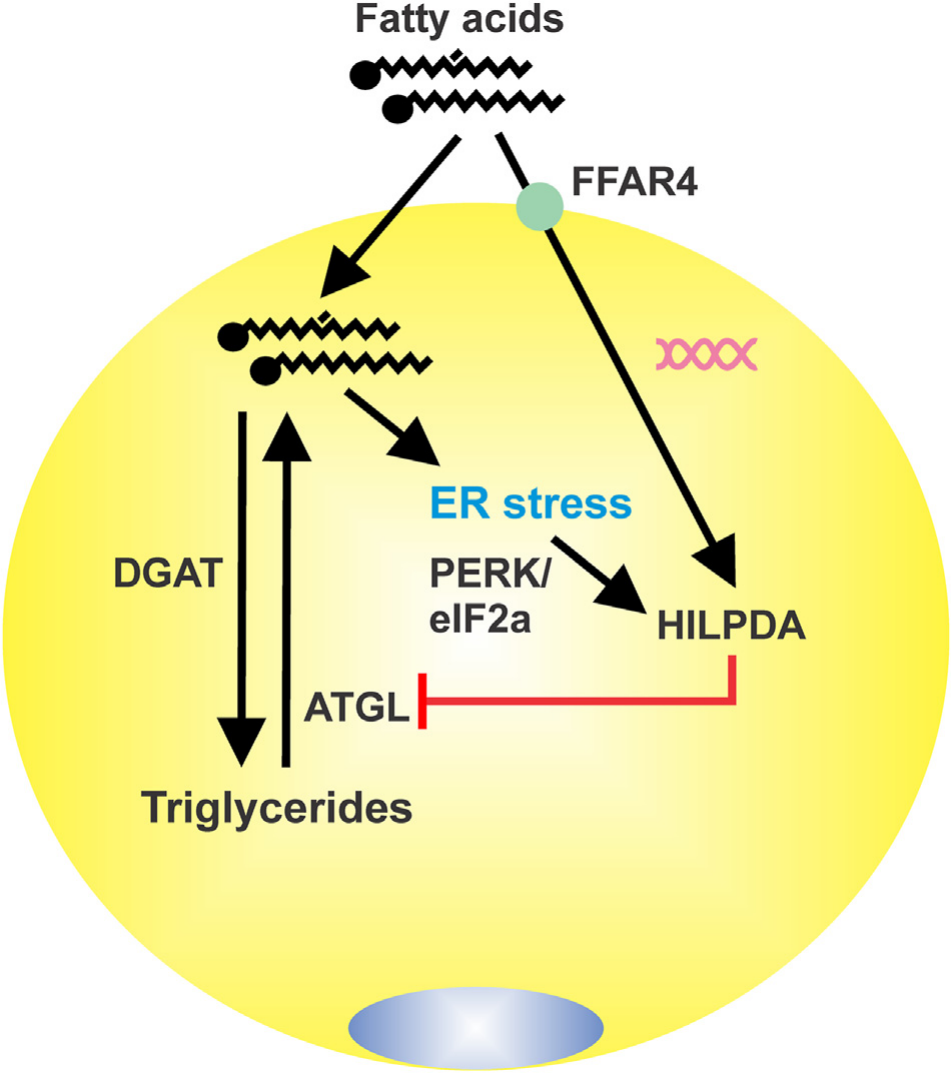
**Graphical depiction of the autocrine negative feedback regulation of triglyceride hydrolysis by fatty acids in adipocytes**. Extracellular fatty acids upregulate *Hilpda* mRNA at least partly via FFAR4. Intracellular fatty acids upregulate HILPA protein at least partly by inducing ER stress and the PERK/eIF2α branch of the UPR pathway. Elevated HILPDA inhibits intracellular lipolysis by downregulating ATGL protein levels.

Here we show that in adipocytes, the excessive elevation of extra- and intracellular fatty acids triggers a feedback suppression of ATGL-catalyzed triglyceride hydrolysis via the induction of HILPDA (Figure 8). By downregulating ATGL protein levels, HILPDA represses lipolysis and aims to restore homeostatic fatty acid control. Extracellular fatty acids upregulate HILPDA levels at least in part via the fatty acid receptor FFAR4, whereas elevation of intracellular fatty acid levels raises HILPDA levels mainly via induction of ER stress and subsequent activation of the PERK/eIF2a branch of the UPR pathway. When HILPDA is deficient, the suppression of ATGL-catalyzed triglyceride hydrolysis by fatty acids is diminished, leading to enhanced fatty acid-induced ER stress when fatty acid re-esterification is disrupted or adipocytes are exposed to excessive levels of fatty acids. Overall, our data suggest that HILPDA is a key player in the negative feedback regulation of intracellular lipolysis in adipocytes by fatty acids, thereby protecting against the lipotoxic effects of fatty acid overload.

Evidence abounds indicating that HILPDA lowers ATGL activity and associated lipolysis in various cell types [19,20,22,[25], [26], [27]]. Biochemical studies, however, have shown that despite the clear physical interaction between HILPDA and ATGL, HILPDA is only a weak direct inhibitor of ATGL activity, in particular when compared with the related protein G0S2 [19]. In our studies, we found that HILPDA deficiency increases ATGL protein levels, suggesting that HILPDA downregulates ATGL protein levels. These data are consistent with other studies showing that 1) adenoviral-mediated overexpression of HILPDA reduces ATGL protein levels in 3T3-L1 adipocytes, and 2) HILPDA deficiency is accompanied by an increase in ATGL protein in macrophages treated with LPS or fatty acids [25,26,28]. The suppression of ATGL protein levels by HILPDA in LPS-treated macrophages was attributed to enhanced proteasomal ATGL degradation [25]. In contrast, here we found that in adipocytes, ATGL is not degraded via the proteasomal pathway. Furthermore, it was observed that proteasomal inhibition lowers ATGL protein levels by raising HILPDA protein levels. Although strictly we cannot rule out that the effect of HILPDA on ATGL protein levels does not require direct physical interaction between the two proteins, given the extensive evidence supporting a direct physical interaction, we consider this scenario unlikely. Further research is necessary to determine how exactly ATGL is degraded in adipocytes and how this process is influenced by the interaction with HILPDA.

Exposure of various cell types to excessive concentrations of fatty acids causes cell stress and ER stress and triggers apoptosis [39,40]. These lipotoxic effects are observed with long-chain saturated fatty acids, such as palmitate and stearate, and to a much lesser extent with unsaturated fatty acids, such as oleate [[41], [42], [43], [44], [45], [46]]. Fatty acid-induced toxicity has been mainly studied in pancreatic beta cells, (cardio)myocytes, hepatocytes, and macrophages, which are cells that may exhibit ectopic fat accumulation in obesity [39,40]. Although due to their large lipid buffering capacity, adipocytes might be expected to be refractory to the toxic effects of fatty acids, they too exhibit ER stress and apoptosis, albeit at higher concentrations of fatty acids than other cell types [47,48]. We found that incubation of adipocytes with OA:PA was able to induce ER stress but only at a very high concentration (900 μM). Inhibition of DGAT-mediated fatty acid re-esterification also caused ER stress in adipocytes, which was abolished by ATGL inhibition and attenuated by HILPDA overexpression. By contrast, HILPDA deficiency enhanced ER stress elicited by DGAT inhibition. Inasmuch as *Hilpda*^ΔADIPO^ adipocytes exhibited residual HILPDA expression, our results may have underestimated the impact of HILPDA on ER stress markers. Overall, the data suggest that under conditions of fatty acid overload, adipocytes attempt to maintain intracellular fatty acid homeostasis and prevent lipotoxicity by upregulating HILPDA and suppressing ATGL-mediated lipolysis.

Similar to cancer cells, macrophages, and hepatocytes [21,24,26,27], we find that external fatty acids also upregulate HILPDA protein levels in adipocytes, which was mediated at least partly via FFAR4. Fatty acids were previously shown to exert feedback inhibition on adipocyte lipolysis by activating FFAR4 and suppressing cAMP levels [[30], [31], [32], [33]]. Fatty acids were also shown to promote lipid droplet formation in Huh-7 hepatoma cells by activating FFAR4, which initially is independent of exogenous lipid uptake [49]. Our data suggest that HILPDA may mediate the autocrine negative feedback regulation of adipocyte lipolysis and the early stimulation of lipid droplet accumulation by fatty acids via FFAR4. According to this model, extracellular fatty acids, possibly released by lipolysis, activate FFAR4, leading to upregulation of HILPDA. Increased HILPDA in turn suppresses ATGL protein levels, thereby decreasing ATGL-catalyzed lipolysis and promoting lipid droplet accumulation.

Besides via extracellular fatty acids, HILPDA protein levels in adipocytes were also strongly induced by intracellular fatty acids, which was at least partly mediated by ER stress and subsequent activation of UPR. Consistent with this notion, HILPDA protein levels were potently stimulated by the ER stressor Thapsigargin. The UPR involves three stress sensor proteins: inositol-requiring enzyme 1α (IRE1α), activating transcription factor 6 (ATF6), and protein kinase R (PKR)-like endoplasmic reticulum kinase (PERK, EIF2AK3), the latter of which phosphorylates the eukaryotic Initiation Factor 2 alpha (eIF2α). Chemical inhibition of the PERK pathway markedly blunted the increase in HILPDA protein levels by DGAT inhibition, suggesting that intracellular fatty acid accumulation increases HILPDA protein via the PERK/eIF2α/ATF4 branch of the UPR. Based on the strong induction of HILPDA by Thapsigargin and fatty acids via the UPR pathway, HILPDA might be considered a marker for lipotoxicity and ER stress in adipocytes.

In the present paper, adipose ATGL levels and ER stress markers were elevated in HILDPA-deficient mice in the refed state, while plasma glycerol was specifically elevated in HILDPA-deficient mice in the fasted state. It is difficult to provide a coherent explanation for these observations, but it could be speculated that the different measurements have different kinetics. Possibly, the lipotoxic response to increased lipolysis in fasted HILPDA-deficient mice—as reflected by higher plasma glycerol levels—may be delayed and only become detectable after several hours of refeeding, when insulin and other metabolic factors go up and differences in plasma glycerol between wildtype and HILPDA-deficient mice are abolished. The reason why HILPDA impacted adipose ATGL levels only in the refed state is unclear. Although ATGL activity is higher in the fasted state, fatty acid overload may also occur in the refed state, necessitating the role of HILPDA to suppress lipolysis and activate fatty acid esterification. Mechanistically, it could be hypothesized that the interaction between HILPDA and ATGL is modulated by an additional factor, such as CGI-58/ABHD5, that is dependent on nutritional status. Another intriguing observation is that HILPDA mRNA and protein levels were similar in adipose tissue of fasted and refed wildtype mice, whereas previous data indicated that adipose HILPDA levels are increased by fasting when compared to the ad libitum fed state [28]. This apparent discrepancy may be explained by the fact that the refed state is markedly different hormonally and metabolically from the ad libitum fed state.

Previously, adipocyte-specific HILPDA deficiency was found to be associated with a reduced weight of the gonadal fat depot after high-fat feeding, an effect that was lost at thermoneutrality [29]. In support of these findings, we observed a significant decrease in the weight of various adipose depots in the *Hilpda*^ΔADIPO^ mice compared to *Hilpda*^flox/flox^ mice after fasting and fasting/refeeding. Intriguingly, we did not observe a significant difference in the weight of various adipose depots between *Hilpda*^ΔADIPO^ and *Hilpda*^flox/flox^ mice after either low- or high-fat feeding, nor did we previously see a change in the weight of the epididymal fat depot after a 24 h fast [28]. The reason for these ostensibly inconsistent observations is not clear.

Consistent with the suppression of ATGL-mediated lipolysis by HILPDA, adipocyte-specific HILPDA deficiency resulted in modestly elevated plasma glycerol levels in fasted mice and mice fed a high-fat diet. By contrast, no effect of adipocyte-specific HILPDA deficiency was observed on plasma NEFA levels. In earlier studies, adipocyte-specific HILPDA deficiency did not impact plasma NEFA and glycerol under any of the conditions examined, including fasting, cold exposure, and CL316,243 injection [28]. One possible explanation for the different impact of HILPDA deficiency on plasma glycerol levels between the current and previous studies is a difference in the genetic background of the mice. All studies in the present manuscript were performed using mice (littermates) that had been backcrossed on the C57BL/6J background at least 5 times. Taking into consideration the limited magnitude of the observed effect of HILPDA deficiency on plasma glycerol, we favor the conclusion that in live mice, HILPDA is only a minor physiological regulator of lipolysis in adipose tissue. Rather, as revealed by the studies in cultured adipocytes, HILPDA seems to be much more important under conditions of non-physiological fatty acid overload, for instance, when fatty acid esterification is genetically or chemically inhibited. Accordingly, it would be of great interest to study the impact of HILPDA deficiency under in vivo conditions of fatty acid overload and lipotoxicity, such as the adipocyte-specific DGAT1 deficient mouse model [7]. Alternatively, it is conceivable that the incomplete disappearance of HILPDA protein in *Hilpda*^ΔADIPO^ mice limits the impact of HILPDA deficiency on numerous metabolic parameters, including plasma NEFA and glycerol levels.

In conclusion, we show that HILPDA is a central node in a fatty acid-induced autocrine feedback loop in adipocytes that aims to restrict intracellular triglyceride hydrolysis under conditions of excessive intra- or extracellular fatty acids to maintain lipid homeostasis and prevent lipotoxicity.

## Declaration of competing interest

The authors declare that they have no known competing financial interests or personal relationships that could have appeared to influence the work reported in this paper.

## Data Availability

Data will be made available on request.
